# Case report of a child with influenza and fatal community-associated methicillin-resistant *Staphylococcus aureus* sepsis

**DOI:** 10.1590/0037-8682-0050-2020

**Published:** 2020-06-22

**Authors:** Bruno Cruz Boettger, Thais Freitas Teles Rezende, Nathalia Bibiana Teixeira, Antonio Carlos Campos Pignatari, Carlos Roberto Veiga Kiffer

**Affiliations:** 1 Universidade Federal de São Paulo, Departamento de Medicina, Laboratório Especial de Microbiologia Clinica, São Paulo, SP, Brasil.; 2 Universidade Estadual Paulista, Instituto de Biociências, Departamento de Microbiologia e Imunologia, Botucatu, SP, Brasil.

**Keywords:** Staphylococcus aureus, Methicillin resistance, Influenza virus

## Abstract

In the present study, we report the incidence of septic shock syndrome associated with methicillin*-*resistant *Staphylococcus aureus* in a child who initially presented influenza-like illness and developed septic shock shortly after 48 h of hospitalization, and eventually died within a few hours of the onset of sepsis. *S. aureus* isolated from the blood culture was characterized as the community-associated strain carrying the staphylococcal cassette chromosome *mec* (SCC*mec)* type IV element. Therefore, it is important to better understand the community-associated methicillin-resistant *Staphylococcus aureus* (CA-MRSA) infections and their potential association with influenza for early diagnosis and successful treatment of this fatal disease.

## INTRODUCTION

Infections caused by methicillin-resistant *Staphylococcus aureus* (MRSA) strains are mostly associated with hospital environments, and are generally caused during the course of hospitalizations for more than 48 h^1^. However, in the last few decades, MRSA cases in patients without hospital exposure have also been reported. These cases have been widely categorized as community-associated methicillin-resistant *S. aureus* (CA-MRSA) infections, which are currently well-described worldwide^2^. Despite previous reports of occurrence of such cases in Brazil in general and São Paulo in particular, it has been considered as a low prevalence region for CA-MRSA infections^3^.

*S. aureus* is a part of human microbiota and a common colonizer with prevalence ranging from 11 to 43 % in different regions^2^. Breakage of the skin barrier and/or decreased immunity have been associated with *S. aureus*-related diseases. Influenza is also considered as a known risk factor for staphylococcal diseases^4^. The association of both these infections was reported as early as in the 1930’s, and was described as fulminating pneumonia cases^3^. Therefore, co-infection with influenza virus and *S. aureus* is related to severe illness and death, and this co-infection might be on a rise in certain regions of the world^4^ *.*

In Brazil, little is known regarding the incidence of CA-MRSA, and even much less is known about the lethality of *S. aureus* and influenza co-infection. In the present study, we report a fatal case of a child who developed sepsis and septic shock due to methicillin-resistant *S. aureus* infection followed by influenza infection shortly after 48 h of hospitalization, which led to death within a few hours after the onset of sepsis.

## CASE REPORT

A 21-month-old child who was previously considered a wheezing infant, was admitted to a hospital in the greater São Paulo area on the night of January 28, 2018, following a febrile seizure, and was observed and evaluated for possible etiology. The child was adopted at the age of 12 months with no previous medical history. In the initial tests performed at the hospital, no underlying disease was detected. The patient was doing well and presented only two additional episodes of fever during the first 24 h of hospitalization. Additional investigation performed included a respiratory viral panel assessment (multiplex immunochromatographic assay) and a chest radiograph. On the following day (i.e. on January 30), the results indicated a leukocyte count of 4,340 cells/mm^3^, the chest radiograph showed minor atelectasis, and the viral panel was positive for influenza A virus. Oseltamivir was administered at a dose of 30 mg orally after every 12 h. During the day, the patient presented irritability and drowsiness, lack of appetite, and intermittent fever. Physical examination revealed the appearance of minor bilateral palpebral edema, hyperemia at the site of the peripheral IV line, and diffuse mild exanthematous rash. Later on the same day (January 30) at around 5:00 PM, the patient’s clinical condition deteriorated, with tachycardia and dyspnea. Subsequently, a presumptive diagnosis of sepsis with pulmonary focus was established. The sepsis protocol was initiated, starting with blood withdrawal for cultures at around 5:00 PM on the 30^th^ of January (less than 48 h of hospitalization). Ceftriaxone and clarithromycin were administered, with subsequent referral to an intensive care unit (ICU). Decreased urinary output, pulmonary congestion, atelectasis, and pleural effusion were noted and general life support measures, including orotracheal tube, central venous catheter insertion, and ventilatory support were provided and vasopressor drugs were administered. Further blood test revealed leukocyte count of 3,470 cells/mm^3^. Despite the measures taken, the patient’s condition deteriorated progressively, with excessive bleeding through the catheter insertion, no response to volume expansion and vasopressor drugs, sustained shock, and impaired ventilatory condition with massive pulmonary bleeding through the orotracheal tube, leading to death at around midnight on the same day (January 30).

On the following day after the patient’s death, blood cultures were found to be positive for *S. aureus*. The clinical care team was surprised with these results, and later on the following day (i.e. on January 31), methicillin resistant strain was confirmed by the routine automated antibiogram of VITEK® 2 (bioMérieux, Marcy l'Etoile, France), showing susceptibility to both, cotrimoxazol and clindamycin. Initially, the hospital control team interpreted it as a hospital-acquired MRSA infection associated with the peripheral phlebitis in the IV line insertion site, and suspected it to be the major cause of the infection. However, the aggressiveness of the disease, which clearly suggested toxic shock syndrome, and cotrimoxazole and clindamycin susceptibility, raised the doubt and thus the medical team sent the sample to a research laboratory (LEMC- Laboratory especial of Microbiology Clinic).

 The *S. aureus* isolate from the patient was subjected to molecular characterization, first through real-time polymerase chain reaction (PCR) for the detection of *mec*A gene, and then through multiplex PCR to determine the staphylococcal cassette chromosome *mec* (SCC*mec*) element type^5^. The SCC*mec* type IV was identified, and subsequently, additional PCRs for the detection of other virulence genes were performed. The strain was found to be positive for intercellular adhesion A (*ica*A), intercellular adhesion B (*ica*B), intercellular adhesion D (*ica*D), staphylococcal enterotoxin-like O (*Sei*O), α-toxin (*hla*), and β-toxin (*hlb*), and negative for Panton-Valentine leukocidin (*PVL*) and toxic shock syndrome toxin 1 (*TSST*-1). 

## DISCUSSION

The SCC*mec* type IV element is found mainly in the isolates of community origin (CA-MRSA). CA-MRSAs differs from the hospital-associated MRSAs (HA-MRSAs) in their antimicrobial susceptibility pattern and virulence profile. *S. aureus* virulence genes have also been associated with severe septic and toxic conditions.

The PVL gene is frequently identified in CA-MRSA isolates as one of the main virulence genes. However, several CA-MRSA isolates that cause severe infections do not possess this gene^6^. In these situations, other virulence genes might be responsible for the severe clinical presentations, although their exact mechanisms of action are still not fully understood^6^

Co-infection of influenza virus and *S. aureus* is known to occur for a while now, and many deaths have been attributed to these severe co-occurrences^7^. The relationship between these two microorganisms is still poorly understood, but an *in vivo* animal model (rats) demonstrated the synergism between these two pathogens. Thus, it is believed that influenza can facilitate bacterial infection due to several factors, such as destruction of the respiratory epithelium, which further promotes bacterial invasion and reduction of phagocytic activity of neutrophils, thereby resulting in less efficient bacterial clearance^8^.

In the present case study, the most surprising element was the association of flu with a hypervirulent CA-MRSA isolate. Methicillin resistance was initially unsuspected due the low prevalence of such reports in this region^3^, and the administered antimicrobials were expected to have an adequate spectrum for the suspected pathogens, such as *S. pneumoniae*, *H. influenzae*, and *S. aureus* (with methicillin susceptibility). Virulence factors and severe diseases have been associated with CA-MRSA in several other regions^7^. Thus, toxic shock, although possible, was relatively unexpected in the present case. Although the patient was kept in a safe environment as much as possible, and the support measures and therapy were quickly initiated, the outcome was tragic. 

In conclusion, this report raises awareness regarding the CA-MRSA infection with fatal outcomes and about the possible hypervirulent strains that might be circulating in children or other susceptible populations in certain regions of the world. It also reinforces the importance of attention that needs to be paid during the season of influenza, and to alert the medical community regarding MRSA, a pathogen associated with high mortality. Finally, since CA-MRSA prevalence is recorded as below 10 % in this region^3^, a question must be raised concerning “when the initial empiric antimicrobial therapy should be modified to cover MRSA as well as other resistant pathogens?”. Therefore, surveillance studies are required in this region and should cover the major resistance threats, particularly CA-MRSA.
